# A fatal case of *Aeromonas jandaei* necrotizing fasciitis

**DOI:** 10.1099/acmi.0.000636.v4

**Published:** 2023-10-26

**Authors:** Anjali Anil, Mani Bhushan Kumar, Sachin Chauhan, Pallab Ray, Divya Dahiya, Archana Angrup

**Affiliations:** ^1^​ Department of Medical Microbiology, Post Graduate Institute of Medical Education and Research (PGIMER), Chandigarh, India; ^2^​ Department of General Surgery, Post Graduate Institute of Medical Education and Research (PGIMER), Chandigarh, India

**Keywords:** *Aeromonas jandaei*, anaemia, cellulitis, necrotizing fasciitis

## Abstract

**Introduction.:**

Necrotizing soft tissue infections (NSTIs) are associated with a fulminating course because of their rapid destruction of tissue planes underlying the skin. *Aeromonas *-associated monomicrobial NSTIs are usually associated with exposure to fresh water, particularly among agricultural workers and fish handlers. Albeit uncommon in incidence, urgent medical and surgical intervention are required once a diagnosis has been made.

**Case report.:**

A 40-year-old male patient, a known case of alcoholic liver disease, presented to the emergency department with pain and diffuse swelling of bilateral lower limbs, which quickly progressed to form blackish discolouration and blebs. Blood for preliminary haematological and biochemical investigations, as well as fluid draining from blebs, were sent for microbiological investigation. The Gram stain revealed occasional neutrophils and Gram-negative bacilli, and pure growth in aerobic culture was identified as *Aeromonas jandaei* by matrix-assisted laser desorption/ionization time-of-flight mass spectrometry (MALDI-TOF MS). The patient was started on empirical antimicrobials, although lesions continued to progress and he ultimately succumbed within 12 h of hospital admission.

**Conclusion.:**

As appropriate antimicrobial therapy and early surgical intervention are required for management of the same, occupational exposure and the fulminant course should raise suspicion of *Aeromonas *-associated infections.

## Data Summary

No data were generated during this research or are required for the work to be reproduced.

## Case report

A 40- year-old male, a farmer by occupation, was referred to our centre from a state medical college for progressive cellulitis of the lower limbs. He had presented to emergency in an ambulatory state with complaints of intense pain and swelling in both lower limbs for 2 days. The pain was acute in onset, diffuse over both lower limbs, non-radiating, and not relieved by analgesics. The swelling was diffuse in nature involving both lower limbs, with the presence of tense and glossy skin. He did not have history of fever, shortness of breath, cough, trauma, insect or animal bite. Two years previously, he had been diagnosed with alcoholic cirrhosis with variceal bleeding and managed by endoscopic variceal ligation in our institute.

In the present episode with a diagnosis of cellulitis with doubtful sepsis, he was managed initially at the state medical college with analgesics along with amoxicillin–clavulanic acid. He was tachycardic with a pulse rate of 130 beats min^−1^ and normotensive. His initial laboratory investigations showed haemoglobin of 7 g dl^−1^ (normal range: 12–16 g dl^−1^), severe leukopenia [total leukocyte count (TLC): 900 cells µl^−1^; normal range: 4500–11000 cells µl^−1^) and thrombocytopenia (31 000 µl^−1^, normal range: 150 000 to 450 000 µl^−1^). He was referred to our institute within 24 h of his stay.

**Fig. 1.**
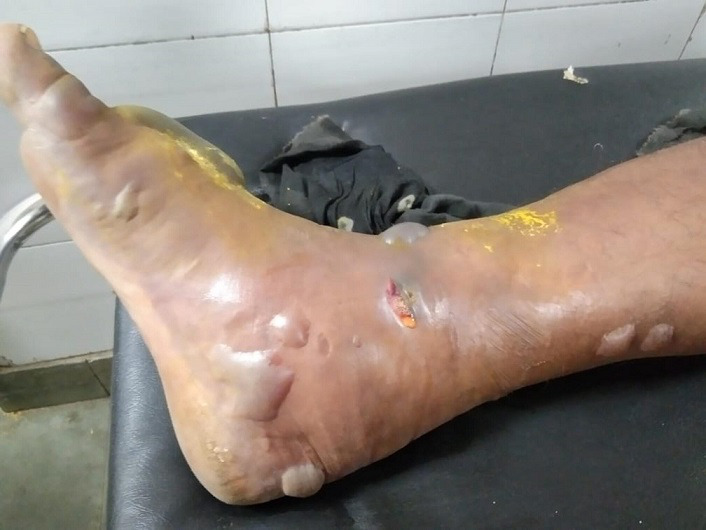
Multiple fluid-filled blebs over the right lower limb.

During his stay in our centre, he developed blackish discolouration of the right lower limb, progressing to fluid-filled blebs (Fig. 1). Following this, the patient also developed blackish colouration and blebs over the left lower limb. Within a few hours, he developed similar features over his right shoulder, although there was no previous oedema over this area. Lesions over the lower limbs quickly progressed to involve the groin and lower abdominal region. In view of worsening respiratory distress, he was intubated. His repeat laboratory investigations at our centre showed haemoglobin of 6.8 g dl^−1^, TLC of 2800 cells µl^−1^ and a platelet count of 25 000 cells µl^−1^, and a differential leukocyte count (DLC) revealed a predominantly neutrophilic picture (80.5 % neutrophils). Coagulation studies revealed prolonged prothrombin time and activated partial thromboplastin clotting time. Renal function (creatinine 2.56 mg dl^−1^) and liver function were deranged, with hyperbilirubinemia and transaminitis. Fluid draining from the blebs was sent to the emergency microbiology laboratory for Gram stain and culture. Gram stain showed Gram-negative bacilli with minimal neutrophilic reaction. The patient was empirically started on intravenous imipenem 1 g 12 hourly and clindamycin 600 mg 8 hourly along with analgesics. However, he developed refractory septic shock and succumbed to his illness within 12 h of admission.

The sample was inoculated on two blood agar plates (aerobic and anaerobic), MacConkey agar and Robertson cooked meat broth (RCM). After overnight aerobic incubation at 37 °C, the blood agar plate showed pure growth of large, smooth, haemolytic, oxidase-positive colonies. The MacConkey agar showed growth of non-lactose-fermenting colonies. No growth of any obligate anaerobe was seen. The isolate was identified by matrix-assisted laser desorption/ionization time-of-flight mass spectrometry (MALDI-TOF MS) (bioMérieux) as *Aeromonas jandaei* with a confidence value of 99.9 %. The isolate was subjected to antimicrobial susceptibility testing by VITEK 2 and reported as sensitive to amikacin, ceftazidime, meropenem, ciprofloxacin and levofloxacin, intermediately sensitive to cefoperazone–sulbactam and resistant to cefotaxime, cefepime, imipenem, piperacillin–tazobactam, tobramycin and aztreonam.

## Discussion

This case report describes a rare monomicrobial necrotizing soft tissue infection by *Aeromonas jandaei *, with a rapidly progressive fatal course, in a patient with alcoholic liver disease.

First described in the 5th Century BCE by Hippocrates, necrotizing soft tissue infections (NSTIs) have been ranked among the most fulminant of infections [1]. They are known to quickly spread to deep tissue planes, although their origin may lie in superficial epidermis or dermis. Clinically, a patient presents with pain out of proportion, cutaneous erythema and necrosis of skin and bullae, with or without systemic signs of toxicity [1].

Although surgical and supportive management depends on the general condition of the patient, knowledge of the causative pathogen and its antimicrobial susceptibility would have a significant role in the targeted treatment of the causative bacteria and their possible toxin production. Currently, a wide spectrum of facultative and obligate anaerobes as well as fungi are implicated. Hence, NSTIs have been classified as types I, II, III and IV based on their respective causative pathogens [2]. Type I, described as polymicrobial NSTI, includes a mixture of facultative anaerobes belonging to the order Enterobacterales, and obligate anaerobes such as *Bacteroides, Clostridium, Fusobacterium* and *Peptostreptococcus *. Type II is typically caused by group A *Streptococcus* and other β-haemolytic streptococci, as well as *Staphylococcus aureus *. Type III includes monomicrobial infections by rare pathogens such as *Aeromonas* species and *Vibrio vulnificus *. [2, 3] They are reported to produce necrotizing fasciitis with sepsis in patients with underlying liver disease, diabetes mellitus, chronic kidney disease, adrenal insufficiency, haematological malignancies and other immunocompromised conditions [4, 5]. According to this classification, this case belongs to type III NSTIs. This patient was a known case of alcoholic liver disease. Lastly, type IV includes fungal pathogens such as *Candida* spp. and Mucorales.

*Aeromonas* species are ubiquitous in the natural environment, and are usually found in fresh or brackish water, sewage, soil, non-faecal organic material and even food sources, particularly seafood [5, 6]. Their virulence is multifactorial and not completely understood. They are capable of producing a multitude of exotoxins (haemolysins, cytotoxins, enterotoxins), haemagglutinins, adhesins (pili, flagella, lipopolysaccharides, capsules) and hydrolytic enzymes such as proteases, enolase, lipases and nucleases. These assist in colonization and ultimately could lead to invasion of tissue [6, 7].

They can potentially cause a spectrum of infections, including gastroenteritis and extra-intestinal manifestations such as wound infections, aspiration pneumonia, sepsis and rarely meningitis and urinary tract infections [6]. Previously, *Aeromonas* species have also been reported to cause polymicrobial necrotizing infections, along with other Gram-negative aerobes and anaerobes [5, 8]. However, monomicrobial infection of *Aeromonas* species has been described as a significant mortality indicator, as per an 18 year retrospective study performed in Taiwan, RC on cases of necrotizing fasciitis in *Aeromonas* [4]. Our patient also had culture-proven monomicrobial infection by *A. jandaei *. The mode of acquisition includes exposure to sources of water or organic matter via trauma, injuries during sports activities, trivial abrasions and reptile bites [4, 9]. Occupational exposure can occur in farmers and fish handlers [5]. In this case, the patient was a farmer by occupation. However, he was not able to recall exposure to stagnant water, bite of insects or reptiles. Commonly implicated species in NSTIs include *Aeromonas hydrophila, Aeromonas caviae* and *Aeromonas veronii* biovar sobria in developed as well as developing countries. Among the species described under the genus, *A. jandaei* has been reported to be rare in clinical specimens [4, 6].

Clinical presentation of *Aeromonas* wound infections can range from non-specific deep-seated pain to fulminant cellulitis and necrotizing myositis. Skin lesions can appear as haemorrhagic bullae, subcutaneous bleeding, purpura, or in the form of gangrene, and these are notorious for causing confusion among clinicians. In our case, the patient showed features suggestive of muscular necrosis with a likely systemic spread, as indicated by the rapid involvement of multiple limbs. Huang *et al*. reported a similar case, caused by *A. hydrophila *, which progressed quickly to involve bilateral lower limbs, followed by upper limbs. As in our case, the patient rapidly progressed to a state of septic shock and died within 12 h of admission [10].

The incidence of *Aeromonas* bacteraemia ranges from 0.12–3.3% and the associated mortality rate ranges from 25–30 % [4, 9]. It has a male preponderance and is commonly acquired from the community [9, 11]. Previously, *A. veronii* biovar sobria causing bacteraemia with intravascular gas production and marked intravascular haemolysis has been described [12]. Similar to our patient, this report describes a patient with intense pain and swelling, with no toxic signs on initial presentation. Evidence of possible bacteraemia could not be established in our patient, owing to a lack of blood samples for bacterial culture. However, his coagulation studies were deranged and sepsis leading to disseminated intravascular coagulation was likely.

Hepatic cirrhosis has been implicated as a major risk factor for the severity of disease and mortality in *Aeromonas* infections. The resulting impaired phagocytic activity by the reticuloendothelial system can cause quick progression to septic shock with multiple organ failure [4]. As described in a case series published in 2003, severe complications were associated with patients with *Aeromonas* septicaemia, with underlying hepatic disease [13]. Hypoalbuminemia, hyperbilirubinemia and deranged prothrombin time are also associated with higher risk of mortality [4, 5]. Previously, TLC <1000 cells µl^−1^, anaemia (haemoglobin <10 g dl^−1^) and platelet count <15×10^4^ cells µl^−1^ were found to be more associated with non-survivors of necrotizing fasciitis with *Aeromonas* species [4]. In this case, the patient’s laboratory investigations showed severe anaemia, leukopenia and thrombocytopenia. He also had underlying alcoholic cirrhosis of the liver with hyperbilirubinemia, hypoalbuminemia and abnormal prothrombin time, all leading to substantial morbidity.

As understood by the severity of *Aeromonas* wound infections, it is important that the patient receives timely surgical debridement along with appropriate antimicrobial therapy to prevent limb loss and death. With the exception of a few strains, *Aeromonas* species are usually resistant to ampicillin, amoxicillin and amoxicillin–clavulanate [4, 9]. They are generally found to be susceptible to third- and fourth-generation cephalosporins, carbapenems, fluoroquinolones and aminoglycosides [4, 9]. Cephalosporin resistance has been associated with poor survival outcomes, as in our patient, because empirical management would usually consist of third-or fourth-generation cephalosporins or carbapenems [4]. Delay until surgery of >24 h also showed significantly poor outcomes in *Aeromonas* necrotizing fasciitis [4, 10]. In this case, the patient could not undergo adequate debridement or receive targeted antimicrobial therapy, as resistance to imipenem was found post his demise.
